# Supplementary material for: An Identification System Targeting the SRK Gene for Selecting S-Haplotypes and Self-Compatible Lines in Cabbage
Source: Plants (Basel). 2022 May 21;11(10):1372. doi: 10.3390/plants11101372 (PMC9145907; doi:10.3390/plants11101372)
Supplement: Supplementary file 1 [file plants-11-01372-s001.zip › plants-1694209-supplementary.pdf]

Supplementary Table S1 Self-compatible index of winter cabbage material (2019)

Material Number	Head Shape	Maturity (day)	Code Name	Self-Compatible Index	Self-Compatibility
18-481	Flat-headed	85	Hbian-2	0	IC
18-482	Flat-headed	80	HPL-1246	>2	C
18-485	Flat-headed	75	A302	0	IC
18-487	Flat-headed	70	HN-25	0	IC
18-491	Flat-headed	70	HN-1	0.33	IC
18-493	Flat-headed	75	M3	>2	C
18-494	Flat-headed	80	Spring Xiu	0.13	IC
18-495	Flat-headed	70	DS308	0	IC
18-496	Flat-headed	70	Yinfeng	0.25	IC
18-498	Flat-headed	80	Jiali	0	IC
18-499	Flat-headed	85	HY-26	0.19	IC
18-503	Flat-headed	70	Jiali-647	0	IC
18-505	Flat-headed	90	Jiali-649	0	IC
18-512	Flat-headed	75	Ydong-1	0.12	IC
18-515	Flat-headed	70	11-62	>2	C
18-520	Flat-headed	75	Han General	>2	C
18-522	Flat-headed	80	H180	0	IC
18-523	Flat-headed	85	H183	0	IC
18-524	Flat-headed	80	H177	0	IC
18-525	Flat-headed	75	DS525	0.11	IC
18-527	Flat-headed	70	H175	0	IC
18-528	Flat-headed	85	Kebic-528	0	IC
18-531	Flat-headed	85	Kebic-531	0.50	IC
18-534	Flat-headed	80	New Yinfeng	0	IC
18-536	Flat-headed	85	RG22-536	0	IC
18-542	Flat-headed	85	RG22-542	0	IC
18-549	Round-headed	80	YK-143	0.37	IC
18-551	Round-headed	80	E05-13	0.14	IC
18-552	Round-headed	90	E05-17	0.83	IC
18-554	Flat-headed	75	Han 4	0	IC
18-557	Round-headed	80	Bejo1039	>2	C
18-562	Round-headed	85	bejo1038Q2	>2	C
18-563	Round-headed	90	09-1039Q	0	IC
18-568	Round-headed	85	Amazon 568	0	IC
18-574	Round-headed	85	Amazon 574	0.9	IC
18-579	Flat-headed	85	HB-48	0	IC
18-583	Round-headed	80	10-511	1.70	C
18-585	Round-headed	85	H12-116Q	>2	C
18-588	Round-headed	75	Parte	>2	C
18-591	Round-headed	70	1186-591	0.67	IC
18-599	Round-headed	75	1186-599	0	IC
18-605	Round-headed	75	1186-605	>2	C

18-611	Round-headed	80	JS602	0	IC
18-614	Round-headed	75	S-H101	0.38	IC
18-622	Round-headed	70	Emerald	0	IC
18-637	Round-headed	75	GOLD B90-637	0	IC
18-639	Round-headed	75	GOLD B90-639	0	IC
18-643	Round-headed	80	A1012	>2	C
18-655	Round-headed	65	107-655	0	IC
18-659	Round-headed	60	A221	0	IC
18-664	Round-headed	55	TC-58Q1	0.56	IC
18-665	Round-headed	60	lvY-Q1	0	IC
18-670	Round-headed	70	TC58-618	0	IC
18-675	Round-headed	65	YF675	0	IC
18-684	Round-headed	70	HanChuanNo.1-684	0	IC
18-695	Round-headed	70	HanChuanNo.1-695	0	IC
18-699	Round-headed	80	MiLan	0	IC
18-JY93	Flat-headed	90	JL-JY82	>2	C

"IC" means "self-incompatibility" and "C" means "self-compatibility".

Supplementary Table S2 The results of 58 materials amplification and sequencing comparison

Material Number	KD4/KD7	PKC6F/ PKC6R	S-Haplotype	Compliance with the Phenotype
18-481	-	+	S68	√
18-482	+	-	S15	√
18-485	+	-	S15	×
18-487	-	+	S68	√
18-491	-	+	S68	√
18-493	+	-	S15	√
18-494	+	-	S15	×
18-495	-	+	S6	√
18-496	-	+	S68	√
18-498	-	+	S68	√
18-499	-	+	S12	√
18-503	-	+	S68	√
18-505	-	+	S12	√
18-512	-	+	S68	√
18-515	+	-	S15	√
18-520	+	-	S15	√
18-522	-	+	S6	√
18-523	-	+	S51	√
18-524	-	+	S51	√
18-525	-	+	S68	√
18-527	-	+	S51	√
18-528	-	+	S23	√
18-531	-	+	S68	√
18-534	-	+	S12	√
18-536	-	+	S68	√
18-542	-	+	S68	√
18-549	-	+	S7	√
18-551	-	+	S7	√
18-552	-	+	S7	√
18-554	+	-	S15	×
18-557	+	-	S15	√
18-562	+	-	S15	√
18-563	-	+	S14	√
18-568	-	+	S14	√
18-574	-	+	S14	√
18-579	-	+	S68	√
18-583	+	-	S15	√
18-585	+	-	S15	√
18-588	+	-	S15	√
18-591	-	+	S7	√
18-599	-	+	S33	√

18-605	-	+	S7	×
18-611	-	+	S7	√
18-614	+	-	S15	×
18-622	-	+	S33	√
18-637	-	+	S68	√
18-639	-	+	S68	√
18-643	+	-	S15	√
18-655	-	+	S45	√
18-659	-	+	S45	√
18-664	-	+	S33	√
18-665	-	+	S33	√
18-670	-	+	S33	√
18-675	-	+	S45	√
18-684	-	+	S33	√
18-695	-	+	S45	√
18-699	-	+	S45	√
18-JY93	+	-	S15	√

"-" means no amplification; "+" means amplification; "√" means that the results are consistent; "×" means that the results are inconsistent.

Supplementary Table S3 The combination and results of flowering pollination experiment

Flowering Pollination Combination	Compatible Index	Compatibility
18-512 ⊗	0	IC
18-2169 ⊗	>2	C
18-2170 ⊗	0	IC
18-503*18-2170	0	IC
18-503*18-2169	>2	C

Supplementary Table S4 The eighteen pairs of SSR primers used to study the genetic background similarity of the 512 sister lines.

Primers	Chromosome	Sequence of Primer	Tm (℃)
BoE188	1	F：CGACGATGGCGAGGAAACA	58
		R：CACATAACCCAAATACCCAAATCA	
BoE607	1	F：TCTATTCACAACGATTCAACTAAC	55
		R：CGGTACGGCTGGCTCTT	
BoE162	2	F：AGCAGCTTCGTTCAATCTCC	58
		R：CGGCAGCGTATACCTTCACA	
BoE966	2	F：TCGAATAAAGAAGAAAAAGAAGA	55
		R：TAATCCCTGGTAAGAGTAGT	
BoE222	3	F：ACTACCCTCTCCGTTTACTCCACA	55
		R：GCCCCATAGCTTTCTCAA	
BoE718	3	F：CAAGAAACGGACGTGGTGAAAG	55
		R：TCTCGCGTATGGGGCTGTCT	
BoE002	4	F：CGTCACGGTGGCGCTTTATTTT	58
		R：ACGACGTCGCCGCACTGAAC	
BoE450	4	F：TCTCGCCATGGCTGATAAG	55
		R：TCGGGGCGTTGATTCTCGTCTCT	
BoE882	5	F：CCGCTTCTTCCTTGCCTTCCT	52
		R：TTCGCCAGTAGATCCCCGTAATG	
BoE699	5	F：TCCCCACCCCCAAAAAGAGA	58
		R：AACGAGCCATCCGAAGAAGAGG	
BoE379	6	F：GCGGGGACTCTACCTCTA	52
		R：AGCAGCTCAGCATACAAG	
BoE761	6	F：ACTACCCTCTCCGTTTACTCCACA	55
		R：GCCCCATAGCTTTCTCAA	
BoE723	7	F：CGTTGAGGCCGAGAGTGAGAG	55
		R：ATGGACGCCGGAAATGAGAA	
BoE209	7	F：ATCTATCCCATCCGCTCGTCA	55
		R：AACCCCTATTCGCTTACTCC	
BoE875	7	F：CCGACAATGGCTGGAGTAGG	55
		R：GATAAGCCGGTAGAGCATAAGGAG	
BoE134	8	F：CTCTTATTTCTTGTAGGGCTTTTA	55
		R：CCGTTGGAGATGACTGACTG	
BoE734	8	F：TCATCCAAAGAAATCAGAGG	52
		R：ACAGGGAGAAAGAAAAAGAGA	
BoE051	9	F：GAGTCTTCGTCTTCTTCTTCC	55
		R：AGTCGCCATTATTAACACCTCTA	

Supplementary Table S5 Distribution of S-haplotypes in 58 winter cabbages

S-Haplotype	Total	Percentage
S15	15	25.86%
S68	14	24.14%
S33	6	10.34%
S7	6	10.34%
S45	5	8.62%
S12	3	6.89%
S14	3	5.17%
S51	3	5.17%
S6	2	3.45%

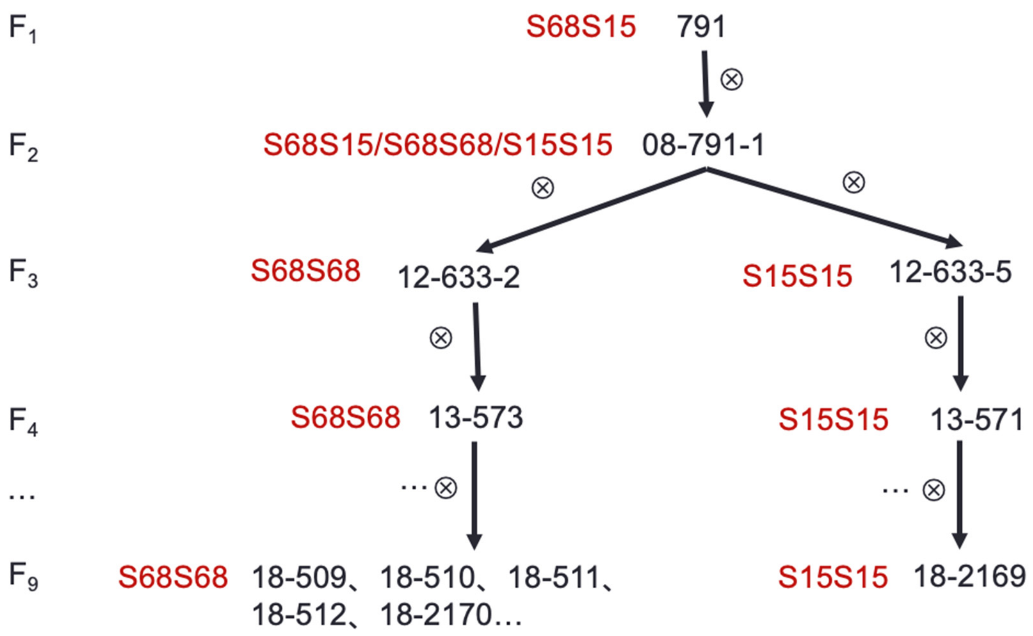

Supplementary Figure S1. The pedigree of the 18-512 sister lines.
